# Cell-Based Models for Development of Antiatherosclerotic Therapies

**DOI:** 10.1155/2017/5198723

**Published:** 2017-02-14

**Authors:** Emile R. Zakiev, Nikita G. Nikiforov, Alexander N. Orekhov

**Affiliations:** ^1^Institute of General Pathology and Pathophysiology, Moscow, Russia; ^2^INSERM UMR_S 1166-ICAN Faculté de Médecine Pitié-Salpêtrière, Paris, France; ^3^Institute for Atherosclerosis Research, Skolkovo Innovation Center, Moscow, Russia

## Abstract

The leading cause of death worldwide is cardiovascular disease. Among the conditions related to the term, the most prominent one is the development of atherosclerotic plaques in the walls of arteries. The situation gets even worse with the fact that the plaque development may stay asymptomatic for a prolonged period of time. When it manifests as a cardiovascular disorder, it is already too late: the unfortunate individual is prescribed with a plethora of synthetic drugs, which are of debatable efficacy in the prevention of atherosclerotic lesions and safety. Cell models could be useful for the purpose of screening substances potentially effective against atherosclerosis progression and effective in reduction of already present plaques. In this overview, we present studies making use of in vitro and ex vivo models of atherosclerosis development that can prove valuable for clinical applications.

## 1. Introduction

Atherosclerosis is a condition resulting in plaque development in the arteries. It has been extensively studied over the last decades. Though the result of this deteriorating process is easily observable via angiography, the causes for the onset of the disease are still debatable. There are several risk factors for the development of atherosclerosis. Improper lipid metabolism, high arterial pressure, and higher-than-normal blood coagulation can all be considered as the possible risk factors. The standard treatment for the atherosclerosis today is to mitigate the symptoms and to hamper further progression. The majority of patients are prescribed with medicines that they have to take indefinitely and which are of debatable safety. The resulting condition may alleviate the symptoms but does not always suggest disease remission, so this is hardly a remedy people desire. Thus, development of a direct antiatherosclerotic therapy is a matter of reversing current plaques and lesions and prevention of the building up of the new ones. Though there was a plethora of large-scale clinical studies on the novel medications, none of them have been proven to possess the properties for effective plaque regression, aspiration of lipid necrotic cores, and stabilization of the fibrous cap at the same time [1–9]. Currently, there are two major groups of antiatherosclerotic treatments, namely, statins and calcium antagonists. Statins are the golden standard in lipid-lowering medications securing low levels of cholesterol level in the blood of patients. Some studies suggest that statins have latent supplementary effects, such as monocyte migration prevention, cell proliferation inhibition, and cellular cholesterol diminution [10–20]. Efficacy of aggressive statin therapy against the atherosclerosis progression has been assessed in multiple clinical trials [9]. The trials of statins have reported various serious adverse effects [21]. This is not to mention that statin treatment is rather expensive [10, 13, 21].

Calcium antagonists are essentially the second major group of interest here [22–30], though there are only four known clinical trials on these substances up to date [9]. Amlodipine, being the most studied of the group, was assessed in intima-medial layer thickness (IMT) reduction in carotid arteries [31, 32]. Other studies on the substance from the group have been proven to be fruitless or with no evidence of clinical efficacy [9].

Some antiatherosclerotic properties were attributed to vitamins-antioxidants, estrogens, and cholesterol/niacin combination, though all results show no efficacy of those substances alone. Estrogen therapy was able to delay the development of atherosclerosis and also slightly turn back time in already existent plaques, but the subject sampling was limited to menopausal females. The results have also been confounded with the influence of ongoing statin treatment, so the efficacy could not have been assessed precisely [33, 34].

**Figure 1:**
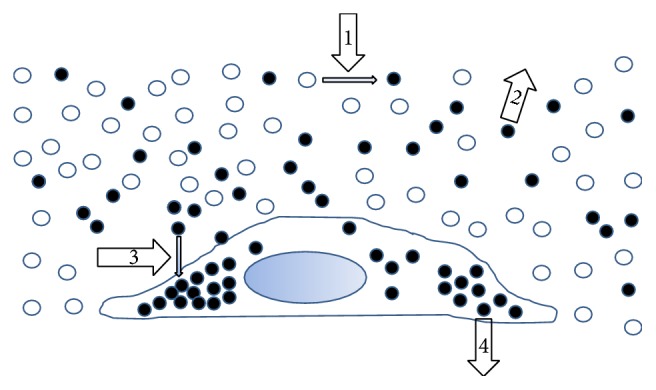
Targets of atherosclerotic and antiatherogenic drug therapy. The figure schematically represents lumen of blood vessel and a vessel wall cell highlighting possible targets for antiatherosclerotic therapy. The first target (target 1) is atherogenic modification (desialylation) of the LDL particle in blood. The prevention of LDL modification may be an approach to antiatherosclerosis therapy. The second approach may be the selective removal of modified LDL from blood (target 2). The third approach may be based on the prevention of modified LDL accumulation in arterial cells (target 3). Additionally, another approach is the removal of excess lipids from foam cells (target 4). Taken with permission from [9].

Naturaceuticals may yield a noticeable therapeutic result with none or mild ill-effects. Possible mechanisms of action for the potentially effective natural drugs are represented on Figure 1. Cell-based models made it possible to test pretty much all of them, using the ability of human serum to provoke atherogenic deteriorations in in vitro and ex vivo cellular models [9]. The cell-based models allow for fast and reliable screening of botanicals potentially efficient in the treatment of atherosclerosis [8]. In this review, we will sum up the studies utilizing cell-based models for seeking new nonpharmaceutical antiatherogenic drugs.

## 2. Cellular Models in the Search for Antiatherosclerosis Therapy

Biological model can be extremely useful in the development of new pharmaceutical and naturaceutical products. Unlike standard laboratory tests for safety and efficacy prior to opening the clinical studies on human population, biological models can be deployed and analyzed quickly. For instance, it is possible to rapidly acquiesce results on activity, metabolism, and direct therapeutic effect of a potential medicine. While keeping the model simple, it is important to retain the likeness to the biological system of interest. It is possible to apply this cellular model strategy to find the naturaceuticals with antiatherosclerotic effect [35–38].

In study [9] authors have deployed two cellular models: in vitro and ex vivo. The in vitro biological model was based on the primary culture of subendothelial cells extracted from thoracic aorta of men and women of 40 to 65 years of age after 1.5–3 hours of their sudden death. The ex vivo model was utilizing the same cell cultures but deployed them in a different way. The in vitro model was aimed to assess botanical and synthetic compounds for their capability of reducing the atherogenicity of human serum as well as to avert the morbid cholesterol accumulation in the intima of blood vessels [7, 9, 28, 30]. Living cells were extracted using collagenase from a variety of regions of aorta, and then they were cultured at 37°C for 7–10 days in an every-day rejuvenated medium [9]. The resulting heterogeneous cell population consisted mostly of pericyte-like cells and typical and modified smooth muscle cells [9]. In order to receive a “control” cellular model possessing different properties from the aforementioned cellular model that was exposed to deteriorations, authors have also taken a healthy region of aorta and cultured cells from it in a similar fashion. Abnormal cellular lipid deposition was induced by incubating the cells with atherogenic serum taken from venous blood of atherosclerotic patients [9]. Products under investigation were added to both of the models, thus testing ability to diminish cellular lipid deposits in already affected cells and the ability to prevent lipid accumulation in relatively “control” cells accordingly. The ability to diminish cellular lipid deposition was tested on the already lipid-laden cells and the antiatherosclerotic effect was regarded as positive if there was a statistically significant decrease of intracellular cholesterol level. The ability to prevent lipid accumulation was measured on the second cellular model, cultivated from normal intimal aorta and to which atherogenic serum was added. The obtained resistance to lipid accumulation was regarded as positive result. In case both stages yielded a positive result, the investigated product was passed on to the next stages of development. The third (ex vivo) model was based on macrophages differentiated from monocytes obtained from venous blood of healthy volunteers by means of culturing for 14 days at 37°C. Volunteers with sufficient blood serum atherogenicity, that is, serum ability to induce the pathological accumulation of cholesterol in the cultivated cell model, were given the investigated product. There was a series of blood collections. The first blood sample was taken prior to the product administration. After the drug administration, blood sample collection was performed after 2, 4, and 6 hours to assess the short-term effects. For the assessment of long-term effects, blood sampling was done after 4, 8, 12, and 24 hours. The serum from those blood samples was added to the culture of aortic cells or monocytes-macrophages. After that, intracellular lipid content, cellular protein content, and calculated serum atherogenic potential were assessed. This mere model allowed authors to assess the antiatherogenic effect of substances taking into account the metabolic processing in the human body. These two models weeded out noneffective botanicals and left only several nonpharmaceutical compounds. Three drugs were developed and taken for further evaluation in the form of clinical studies [8].

## 3. Clinical Trials to Evaluate the Antiatherosclerotic Activity of Developed Drugs

There are three clinical trials that were aimed to test the efficacy and safety of the naturaceutical drugs, screened with cell models. Cultured aortic cell in vitro model was used to find potentially efficient natural agents fighting early-stage atherosclerosis.

### 3.1. Allicor

In this cell-based assay, 31 substances were assessed [39]. The effects of several substances are summarized in Table 1. The majority of investigated substances were pertaining to the flavonoid group. The chemicals under investigation were assessed for cytotoxicity via measurement of the absolute protein ratio and cell viability using trypan blue. Authors have weeded out eight substances with cytotoxic effect, three of which had proatherogenic activity. Then two more substances with proatherogenic activity and four substances with no activity were eliminated, summing up to fourteen substances excluded totally. Authors based their short-list botanical substances on literature studies. Botanicals like spirulina* (Spirulina platensis)*, onion* (Allium cepa)*, wheat germs* (Triticum vulgaris),* hill-growing saltwort* (Salsola collina)*, beetroot* (Beta vulgaris)*, garlic* (Allium sativum)*, licorice* (Glycyrrhiza glabra)*, and extract of pine straw* (Pinus sylvestris)* have been evaluated in this study. These substances were tested in the aforementioned ex vivo model.

Authors used garlic powder in capsules for its effectiveness: six hours after the administration, the baseline rate of atherogenicity was three times lower. Based on these premises, authors have developed a natural-based product called “Allicor.” Clinical study confirmed the antiatherosclerotic effect of Allicor at the level of the vascular wall. The primary endpoint of the clinical trial was the rate of cIMT progression/regression [8]. The subjects in this clinical trial were all men, aged 40–74. 257 individuals were screened and randomized to the study [8]. The inclusion criteria were the following: carotid atherosclerosis; absence of a prolonged treatment (more than 2 months per year) with sugar-lowering drugs, diuretics, and vasoactive medicines; the maximum cIMT of 1-2 mm in order to be detectable during the first B-mode ultrasound examination; systolic blood pressure below 160 mmHg; and diastolic blood pressure below 90 mmHg. 257 subjects were randomized into 2 treatment groups—Allicor treatment group and control group [8]. 196 patients in total were eligible for the assessment of the main endpoint. The primary aim of the trial was to confirm or reject efficiency of Allicor in influencing the rate of cIMT progression/regression. In the treatment group, 32.3% of patients had their cIMT increased, while 47.3% of patients had a significant decrease in cIMT. In the control (placebo) group, 48.5% of subjects had an increase in cIMT, and 30.1% of the subjects had a decrease in cIMT. cIMT dynamics in the treatment group were significantly different, as compared to the control (placebo) group. Result of the trial was that the average cIMT regression in the treatment group was 0.022 ± 0.007 mm per year. The divergence of total cIMT regression rate between control and treatment groups after two years of administration was still detectable and the gain was in favor of the treatment group. Decrease of patients' blood serum ability to cause intracellular cholesterol accretion in the ex vivo cell culture test took place. In the treatment group, serum atherogenicity decreased nearly by 30% from the baseline level in as soon as 3 months after the start of administration, and the effect retained its magnitude till the end of the study, while the placebo group, on the contrary, had no significant changes. Decrease of patients' serum atherogenicity was directly correlated with reduction of cIMT. In either of groups, level of HDL cholesterol has risen significantly during the study, while changes in triglyceride level were not significant [8]. Adverse events distribution was random, and none of the fatal serious adverse events were associated with the treatment. Summing up, antiatherosclerotic effectiveness of Allicor was approved, and cIMT regression induced by Allicor was demonstrated clearly in the study [8]. Allicor successfully passed through clinical trials phases I to III and was recommended as an effective product for long-term therapy of an early stage of atherosclerosis [8].

### 3.2. Inflaminat

Inflammation is a constant companion of vascular regions affected by atherosclerotic lesions [40]. The most important interleukins in atherosclerotic process are IL-6 and IL-1. IL-1 promotes local inflammation, while IL-6 is a proinflammatory factor and an important marker of inflammation in coronary atherosclerotic lesions. Inflammation theory of atherogenesis is extensively tested using in vivo models in order to assess the efficiency of inflammation inhibition in the antiatherosclerotic therapy. Before the occurrence of clinical manifestations, elevated level of inflammation markers can be registered in the blood serum. Current efforts are focused on assessing how important the role of cytokines is in chronic malicious conditions, and a direct anticytokine therapy is under development now. But, for the moment, the majority of the potential anticytokine medications are on the research stages, while those which already went through clinical trials have not shown desired efficacy and specificity. Taking that into account, there are some studies that show efficiency of compounds of natural origin in the inhibition of IL-1, IL-6, and tumor necrosis factor-alpha (TNF-*α*).

Thus, the aim of this research was to create a naturaceutical product possessing anti-inflammatory properties as a supplement in patients with atherosclerosis [41]. Utilizing the same in vitro cellular model as in the screening stage for Allicor (see above), 31 selected botanicals were tested for their anticytokine activity. Of those, only 5 have shown the ability to hamper IL-1 expression, namely, violet, calendula, elder, hawthorn, and St. John's wort.

Authors obtained a combination of three active ingredients (elder, calendula, and violet) which was effective in the inhibition of expression of both IL-1 and TNF-*α*. The product was given the name Inflaminat and it was placed into production at INAT-Pharma, Russia [41]. Inflaminat's activities in anticytokine and antiatherogenic roles were assessed in a series of studies. The comparison with Diclofenac and Allicor was made. Authors recruited healthy volunteers, males and females with 52–69 years of age, whose blood possessed atherogenicity and proinflammatory activity. Once collecting the baseline blood sample, patients were given a dose of investigational product or comparator. After that, blood was taken after 2, 4, and 8 hours. In ex vivo model, Inflaminat was able to significantly decrease the IL-1 concentration for nearly 25% from the baseline level, while for Diclofenac the decrease was 49%. 8 hours after administration, TNF-*α* expression decreased by 9% for Inflaminat and by 39% for Diclofenac. 8 hours after administration, Inflaminat was able to reduce serum atherogenicity by 64% compared to the initial level: Allicor by nearly 50% and Diclofenac by 13%. This way, anticytokine and antiatherogenic properties of Inflaminat were demonstrated. The results were comparable to those for Diclofenac and Allicor. This fact shows the efficacy of the tested drug on biological models and its safety for healthy volunteers [42]. The initial double-blinded randomized placebo-controlled trial was aimed to assess efficacy and safety of Inflaminat in subjects with subclinical carotid atherosclerosis [41, 42].

Clinical trial of Inflaminat included men aged 40–74 with asymptomatic atherosclerosis. Exclusion criteria were the following: cardiovascular or cerebrovascular disease; comorbidity that needed continuous treatment (more than 2 moths per year); signs to surgical interventions of atherosclerosis located in extracranial region of the brachiocephalic system; and individual intolerance to treatment with anti-inflammatory drugs. 78 patients entered the screening period. Systolic blood pressure decreased by 19 mmHg and diastolic arterial blood pressure decreased by 6 mmHg; total cholesterol level decreased by 49 ml/dL and LDL cholesterol decreased by 51 mg/dL in the treatment group. 10-Year prognostic risks for myocardial infarction, sudden cardiac death, and ischemic heart disease decreased by 10%. cIMT of the right common carotid artery decreased by 62 *μ*m in average. At the same time, in the control group, statistically significant decrease of total cholesterol was detected, probably due to diet correction recommendations received by all patients in the beginning of the trial. Nevertheless, LDL cholesterol, triglycerides, atherogenic index, and cIMT have not decreased. The results of the initial double-blinded randomized placebo-controlled clinical trial of Inflaminat approve that Inflaminat has antiatherosclerotic effect and caused a regression of atherosclerotic lesions at the dawn of the disease. Treatment with Inflaminat also had positive effects on different cardiovascular risk factors and caused the lowering of the prognostic risks of ischemic heart disease and myocardial infarction.

### 3.3. Karinat

Atherosclerosis-related pathologies are the main reason of sudden death in women (up to 73%). Nearly 55% of deaths in females are due to cardiovascular pathologies, and this proportion is higher than that in males. So, it turns out that while the major attention was paid to prevention of the atherosclerosis in men, prevention of atherosclerosis in women was left vastly underscored. The new trend in the biology is establishing new therapies for prevention and therapy of atherosclerosis in women [43, 44].

Till now, there has been no effective therapy that would have been able to improve the quality of life and avert atherosclerosis progression in menopausal women at the same time. Hormone replacement therapy is effective against osteoporosis and in alleviating the menopause symptoms but has oncogenic features and even stimulates the cardiovascular disorders.

Natural phytoestrogens bear similarity to human estrogens and have ability to block estrogen receptors [53]. This research [54] aimed to develop a new breed of drugs for menopausal women that would reduce the menopause symptoms and at the same time prevent the onset of atherosclerotic process without serious adverse effects. Authors have searched for the most effective and safe phytoestrogens in terms of positive, satisfactory, or negative antiatherosclerotic effects and pharmacodynamics on in vitro and ex vivo models. Several substances have been weeded out; the rest have been considered as promising, that is, had no detected cytotoxicity and possessed a stable effect. Grape, soybean, sage, carrot, orange, garlic, licorice, onion, hop, green tea, focus, kelp, calendula, clover, hawthorn, elder, and violet were relatively easy to obtain and had high concentrations of the desired substances.

Antiatherosclerotic and antiatherogenic activities of the chosen plants were assessed on the in vitro and ex vivo models. A vast majority of the plants were eliminated due to the lack of activity or absence of long-term effect or low availability. The final list of promising compounds was the following: tannin from grape stone, garlic, hop, sage, and green tea leave. The final formula of active ingredients was proposed in accordance with minimal effective doses of each substance. A product called Karinat was developed. Following the successful laboratory study, the final naturaceutical product was ready for a clinical trial [43, 44, 54]. The initial double-blinded, randomized, placebo-controlled study was conducted in order to evaluate the efficacy and safety of the pills. 131 women were examined during the prescreening period. Exclusion criteria were the following: treatment with hypolipidemic drugs in a period of 6 months prior to the screening; treatment with sugar-lowering drugs for more than 2 months per year, treatment with beta-blockers and calcium antagonists; vestiges of hormone replacement therapy; vestiges of myocardial infarction and/or diagnosed acute cerebrovascular disorders and/or chronic cardiovascular insufficiency and/or pulmonary thromboembolism; the history of carcinoma; uncontrolled arterial hypertension more than 145/95 mmHg; chronic renal insufficiency; hepatic cirrhosis; and individual intolerance to any of Karinat compounds. Totally, 131 females complied with the inclusion criteria. The average age of the participants was 64.8 years. Generally, subjects were having mild overweightness, high-normal arterial blood pressure, mild risk of ischemic disease and myocardial infarction, and subclinical atherosclerosis. Total cholesterol levels decreased in both control and treatment groups, but in the treatment group this decrease was due to LDL cholesterol, while in control group it was due to changes in HDL cholesterol level. In the treatment group, the cIMT increase rate was only 6 *μ*m/year, while in the control group cIMT increase rate was more than 100 *μ*m/year. The placebo group confirms that there is an active atherosclerotic process in postmenopausal women going on. Considering the initial study design and short time span of therapy, none of detected changes in cIMT were considered as statistically significant. The active and aggressive atherosclerosis progression in study participants was also confirmed by the increase of atherosclerotic plaques size. In the control group, the growth rate of already existing plaques topped up to 40% per year, while in the treatment group the growth rate of already existing plagues was 27% per year. The results of the initial double-blinded randomized placebo-controlled clinical trial of Karinat approve the fact that in postmenopausal women Karinat has antiatherosclerotic effect and causes regression of existing atherosclerotic lesions by 1.5 times.

## 4. Conclusions

Atherosclerosis is one of the most important medical and social problems, taking the blame for the development of cardiovascular pathologic conditions, which account for more than half of all death cases. Patients on different stages of the disease have to take the same medications, while an aggressive therapy at the initial stages of the disease may result in adverse events. Currently, the most known antiatherosclerotic drugs are statins. They have been primary developed as drugs lowering serum cholesterol through inhibiting hepatic cholesterol biosynthesis. However, it soon became clear that antiatherosclerotic effects of statins could not be explained entirely by blood cholesterol reduction. Statins possess pleiotropic atheroprotective effects that are independent of cholesterol-lowering [55]. These noncholesterol effects “alter the expression of endothelial nitric oxide synthase, the stability of atherosclerotic plaques, the production of proinflammatory cytokines and reactive oxygen species, the reactivity of platelets, and the development of cardiac hypertrophy and fibrosis” [55].

The exploitation of the concept of serum atherogenicity and the development of the effective cellular models allowed for the evaluation of the “direct antiatherosclerotic” activity of drug substances. As a result of the studies described in this overview, three natural nonpharmaceutical products have been created: Allicor, Karinat, and Inflaminat. All the drugs were tested on healthy subjects and assessed on in vitro and ex vivo models and demonstrated a significant success in decreasing serum atherogenicity. The major outcomes of the treatment were the decrease of cIMT and decrease of intracellular cholesterol accretion. Allicor was assessed in pilot and basic clinical trials, while Karinat and Inflaminat only started their life cycle with pilot studies, and the studies of the latter two are still to be continued.

Table 2 compares the data on the antiatherosclerotic activities of Allicor, Inflaminat, and Karinat with similar data obtained in clinical trials of lipid-lowering drugs. All clinical trials had similar designs. It should be noted that Allicor, Inflaminat, and Karinat as direct antiatherosclerotic drugs realizing their effects at the arterial wall level possessed not less pronounced antiatherosclerotic activity compared to the lipid-lowering drugs that implement their pharmacological effects at the level of cholesterol circulating in blood or having pleiotropic antiatherosclerotic effects [55]. Moreover, Allicor and Inflaminat were as effective or even more effective compared to statins (Table 2). Thus, the drugs developed using our approach have been proven as very effective antiatherosclerotic drugs.

Our approaches are based on the use of in vitro and ex vivo cellular models. The effectiveness of these approaches has been confirmed by the assessment of such surrogate marker of atherosclerosis as a сIMT. Of course, these approaches have their limitations because they do not allow us to estimate the influence of drugs developed on clinical manifestations of atherosclerosis such as myocardial infarction, stroke, and sudden death. This assessment is a challenge for further research. It is not known yet whether the mechanisms of Allicor, Inflaminat, and Karinat antiatherosclerotic actions are implemented via main cardiovascular risk factors or via exclusively alterations in arterial wall. Unlike statins, these drugs are not potent lipid-lowering medications. With regard to Allicor, it was shown that it possesses a mild or moderate hypotensive and cholesterol-lowering effects [40]. Allicor demonstrated antidiabetic and antiviral actions. In men with cerebral atherosclerosis, it has been demonstrated that 14-day treatment inhibited ADP-induced platelet aggregation by 25.4% and increased plasma fibrinolytic activity by 22.4%. One more study was performed in high-risk patients to evaluate the changes of prognostic cardiovascular risk that was calculated using algorithms derived from Framingham and Muenster Studies. Twelve-month treatment lowered 10-year prognostic risk of CHD by 13.2% in men and by 7.1% in women. Ten-year prognostic risk of acute myocardial infarction and sudden coronary death was lowered by 26.1% in men. On the other hand, all three drugs, namely, Allicor, Inflaminat, and Karinat, possess antiatherogenic potential in ex vivo model. There is no doubt that the antiatherogenic effect realizing at a level of the arterial wall makes a substantial contribution to the clinical effects of these drugs.

## Figures and Tables

**Table 1:** Substances tested on cellular models.

Effect	Antiatherogenic	Proatherogenic	Indifferent
Tested substances	Cyclic AMP elevators Prostacyclin Prostaglandin E2 Artificial HDL Antioxidants Calcium antagonists Trapidil and trapidil derivatives Lipoxygenase inhibitors Lipostabil Mushroom extracts	Beta-blockers Thromboxane A2 Phenothiazines	Nitrates Cholestyramine

With permission from [9].

**Table 2:** The comparative data from clinical trials on carotid atherosclerosis regression.

Trial	Medication	Mean annual IMT change, mm		Reference
		Placebo	Treatment	
PLAC II	Pravastatin	0.068	0.059	Crouse III et al., 1995 [45]
KAPS	Pravastatin	0.029	0.010	J. T. Salonen and R. Salonen, 1993 [46]
ASAP	Simvastatin	—	−0.009	Smilde et al., 2001 [47]
PREVENT	Amlodipine	0.011	−0.015	Pitt et al., 2000 [48]
ASAP	Atorvastatin	—	−0.020	Smilde et al., 2001 [47]
CLAS	Colestipol, niacin	0.010	−0.020	Blankenhorn et al., 1993; Hodis, 1995 [49, 50]
MARS	Lovastatin	0.015	−0.028	Blankenhorn et al., 1993; Hodis, 1995 [49, 51]
VHAS	Verapamil	—	−0.028	Zanchetti et al., 1998 [52]
AMAR	Allicor	0.015	−0.022	Orekhov et al., 2013 [8]
NCT01743404	Inflaminat	0.062	−0.068	Orekhov et al., 2013 [8]
NCT01742000	Karinat	0.111	0.006	Orekhov et al., 2013 [8]
